# Comparing the Efficacy of Targeted and Blast Portal Messaging in Message Opening Rate and Anticoagulation Initiation in Patients With Atrial Fibrillation in the Preventing Preventable Strokes Study II: Prospective Cohort Study

**DOI:** 10.2196/49590

**Published:** 2024-01-24

**Authors:** Alok Kapoor, Parth Patel, Soumya Chennupati, Daniel Mbusa, Hammad Sadiq, Sanjeev Rampam, Robert Leung, Megan Miller, Kevin Rivera Vargas, Patrick Fry, Mary Martin Lowe, Christina Catalano, Charles Harrison, John Nicholas Catanzaro, Sybil Crawford, Anne Marie Smith

**Affiliations:** 1 University of Massachusetts Chan Medical School Worcester, MA United States; 2 University of Massachusetts Memorial Health Care Worcester, MA United States; 3 College of Pharmacy University of Florida Jacksonville, FL United States; 4 College of Medicine University of Florida Jacksonville, FL United States; 5 Learning Advisors LLC Chicago, IL United States; 6 Heart Rhythm Society Washington, DC United States

**Keywords:** anticoagulants, atrial fibrillation, humans, outpatients, patient education as topic, patient portals

## Abstract

**Background:**

The gap in anticoagulation use among patients with atrial fibrillation (AF) is a major public health threat. Inadequate patient education contributes to this gap. Patient portal–based messaging linked to educational materials may help bridge this gap, but the most effective messaging approach is unknown.

**Objective:**

This study aims to compare the responsiveness of patients with AF to an AF or anticoagulation educational message between 2 portal messaging approaches: sending messages targeted at patients with upcoming outpatient appointments 1 week before their scheduled appointment (targeted) versus sending messages to all eligible patients in 1 blast, regardless of appointment scheduling status (blast), at 2 different health systems: the University of Massachusetts Chan Medical School (UMass) and the University of Florida College of Medicine-Jacksonville (UFL).

**Methods:**

Using the 2 approaches, we sent patient portal messages to patients with AF and grouped patients by high-risk patients on anticoagulation (group 1), high-risk patients off anticoagulation (group 2), and low-risk patients who may become eligible for anticoagulation in the future (group 3). Risk was classified based on the congestive heart failure, hypertension, age ≥75 years, diabetes mellitus, stroke, vascular disease, age between 65 and 74 years, and sex category (CHA_2_DS_2_-VASc) score. The messages contained a link to the Upbeat website of the Heart Rhythm Society, which displays print and video materials about AF and anticoagulation. We then tracked message opening, review of the website, anticoagulation use, and administered patient surveys across messaging approaches and sites using Epic Systems (Epic Systems Corporation) electronic health record data and Google website traffic analytics. We then conducted chi-square tests to compare potential differences in the proportion of patients opening messages and other evaluation metrics, adjusting for potential confounders. All statistical analyses were performed in SAS (version 9.4; SAS Institute).

**Results:**

We sent 1686 targeted messages and 1450 blast messages. Message opening was significantly higher with the targeted approach for patients on anticoagulation (723/1156, 62.5% vs 382/668, 57.2%; *P=*.005) and trended the same in patients off anticoagulation; subsequent website reviews did not differ by messaging approach. More patients off anticoagulation at baseline started anticoagulation with the targeted approach than the blast approach (adjusted percentage 9.3% vs 2.1%; *P*<.001).

**Conclusions:**

Patients were more responsive in terms of message opening and subsequent anticoagulation initiation with the targeted approach.

## Introduction

About 6 million Americans have atrial fibrillation (AF), with 12 million projected by 2050 [1-3]. AF accounts for 15% of ischemic strokes, resulting in permanent disability in 60% of cases and death in up to 20% [4]. The main approach to stroke prevention is anticoagulation. Although guidelines [5] and evidence exist to guide providers in prescribing anticoagulation, only about 60% of eligible patients receive anticoagulation, leading to a projected annual excess stroke rate of 100,000 [6,7]. Low adherence to this guideline results from a combination of not initiating anticoagulation when indicated and discontinuing anticoagulation prematurely. This is particularly true in patients of minority race and ethnicity, where anticoagulation use is lower and stroke rates are higher [8-13].

There are multiple barriers to initiating and persisting with anticoagulation. Access to specialists, socioeconomic status, and health literacy each represent a barrier [8]. The advent of patient portals makes electronic messaging an attractive, low-cost method to educate patients and prepare them for visits with their anticoagulation providers. While the electronic health record (EHR) patient portal is increasingly being used in health care to improve patient education, engagement, and health outcomes, responsiveness to this methodology for anticoagulation use in patients with atrial fibrillation is unknown.

A recent review suggests that patient education about anticoagulation through a mobile device, such as a smartphone or tablet, increases patient knowledge levels, medication adherence, and satisfaction and is associated with improved clinical outcomes [14]. EHR-based programs have also been identified as a valuable method to improve warfarin therapy, a type of anticoagulation, self-management for pediatric patients with congenital heart diseases [15]. Evaluating patient responsiveness to different portal-based messaging methods can help identify the optimal use of EHR patient portal tools to best support patients in managing their AF and anticoagulation.

In this study, we compare patient responsiveness to 2 approaches to patient portal messaging with the goal of directing patients to the Upbeat website [16] of the Heart Rhythm Society, which contains print and video information about AF and anticoagulation.

## Methods

### Overview

We previously published the protocol for our paper, which covered the methods used at UMass to send patient messages [17]. We will briefly summarize the pertinent elements of the methods for that messaging campaign. We will also include additional details regarding the parallel messaging intervention at the UFL.

### Study Design

We conducted a prospective cohort study. We sent patients a message through MyChart (Epic Systems Corporation), the patient portal associated with Epic Systems (Epic Systems Corporation) EHR, introducing the study and the purpose of communication (Multimedia Appendix 1). The message contained a link (unique to each site) to educational materials housed on a professional society web page—that is, the Upbeat website produced by the Heart Rhythm Society (HRS)—as well as a link to a survey soliciting feedback about the educational materials (Multimedia Appendix 2). Essentially, we created 2 unique websites, (1 for each site) but with the same content and layout (clone copies). In the first approach, at the University of Massachusetts Chan Medical School (UMass), we tested targeted messaging by sending messages to patients through MyChart 1 week before an appointment with a cardiology provider or primary care provider. In the second approach, at the University of Florida College of Medicine-Jacksonville (UFL), we tested a blast messaging approach of sending a message to all eligible patients independent of an appointment. At UMass, we facilitated the message-sending process with a bulk communication tool available through Epic Systems. At UFL, we sent messages manually.

### Setting

We included the cardiology and primary care practices of the UMass Memorial Health System located in central Massachusetts, as well as the patients within UFL’s ambulatory practices located in northern Florida and southern Georgia. Both sites used the Epic Systems EHR and the MyChart patient portal for the duration of the study. We sent messages to UMass patients from November 2021 until February 2022. At UFL, we sent all messages in November 2021.

### Participants

We included patients aged 18 years or older with AF with active MyChart patient portal accounts and who had at least 1 office visit in the 12 months before the start of our messaging intervention in November 2021. At UMass, starting each workday from November 2021 to February 2022, we ran the Epic System’s Reporting Workbench that identified patients based on their having an appointment (office or tele-visit type) with a primary or cardiology care provider scheduled to take place within a week. At UFL, from November 2021 to December 2021, we identified patients based on a previously established registry of those patients with AF who had a visit with a primary or cardiology care provider in the previous year. At both sites, we grouped patients based on their anticoagulation status (eg, on or off anticoagulation) and congestive heart failure, hypertension, age ≥ 75 years, diabetes mellitus, stroke, vascular disease, age 65-74 years, and sex scale (CHA_2_DS_2_-VASc) score. Specifically, group 1 included those at high risk (eg, CHA_2_DS_2_-VASc score of ≥2 for men and ≥3 for women) and currently on anticoagulation; group 2 included those at high risk and off anticoagulation; and group 3 included those at low risk (eg, CHA_2_DS_2_-VASc score <2 for men and <3 for women) and not on anticoagulation.

### Outcomes, Variables, or Data Sources

#### Message Opening

We tracked message opening as the number of messages open divided by the number of messages sent. To identify messages, we relied on Epic Systems clarity structured query language–based coding. Specifically, we collected all messages received from individual patients and then filtered them by messages sent by the study coordinators. Study coordinators did not send messages for other purposes, allowing us to only isolate study-related messaging.

#### Website Review

Using Google Analytics (Google LLC), we tracked the number of unique page views as the value representing the total number of unique sessions. As patients may have received more than 1 message throughout the study (corresponding to 2 separate visits or in the case of canceled and rescheduled visits), we selected the number of messages sent as the denominator. We then calculated the percentage of messages resulting in a unique page view, with the number of unique page views as the numerator and compared this across sites. We also compared the “bounce rate” across sites, which represents the percentage of all sessions on a site in which users only viewed a single page. Google documentation [18] notes that bounce rates should be interpreted within the context of a specific website’s purpose. Upbeat, the website our messages directed patients toward, has many links to educational resources regarding anticoagulation and AF. We consider navigation away from the landing page to indicate more patient engagement with these educational materials. Thus, having a lower bounce rate indicates higher engagement with the Upbeat website beyond the information presented on the landing page. Digital experience research indicates that a bounce rate of less than 40% is excellent [19], although the referenced source did not provide a specific bounce rate for health education websites, which may differ from other types of websites.

#### Survey-Based Outcomes

We compared survey responses across both messaging approaches and sites. For group 1 (high risk, on anticoagulation), the survey covered domains of discussions of personal stroke risk, history of anticoagulation use, and persistence. For group 2 (high risk, off anticoagulation), the survey covered discussions of personal stroke risk, the report by the patient of receiving a provider suggestion to take anticoagulation, and the reason for stopping anticoagulation for those with previous use. For group 3 (low risk, not on anticoagulation), the survey covered the likelihood of learning more about personal stroke risk, willingness to start anticoagulation, and reasons for anticoagulation hesitancy. We also asked all 3 groups of patients about their attitude toward the Upbeat website materials, including if the materials were understandable, useful, and something they would recommend to other patients. We collected responses on a 5-point Likert scale ranging from strongly disagree to strongly agree.

#### Anticoagulation Use After Messaging

We tracked anticoagulation use through medication and laboratory records from our EHR for the 3 months following the completion of our messaging program until May 2023. To be on anticoagulation, a patient had to have an active prescription for an anticoagulant updated at an office visit in the 12 months before the start of the messaging program in November 2021. Moreover, the prescription had to be consistent with a therapeutic dose to prevent strokes associated with AF. We assigned baseline status based on the presence or absence of an anticoagulation medication on the current medication list for a visit occurring in the 12 months before baseline. We also considered a patient to be on anticoagulation if they had an international normalized ratio value of 1.5 or higher recorded within 60 days of the date of the end of follow-up, following an example in the literature as well as the clinical threshold commonly observed to make decisions about surgery and anticoagulation reversal [20,21].

#### Independent Exposure

The independent exposure was the messaging approach used (targeted at UMass vs blast at UFL).

#### Other Exposures: Anticoagulation Outcome Only

We included stroke risk based on the CHA_2_DS_2_-VASc score, which is comprised of congestive heart failure, hypertension, age, diabetes, previous stroke, vascular disease, and gender. To adjust further for potential confounders of the association between anticoagulation use and message opening, we included demographics omitted in that score (ie, race, ethnicity, language preference, and primary insurance). Finally, we included chronic kidney disease and anemia. In general, we relied on the International Classification of Diseases, tenth edition codes for the presence of a comorbid condition. For chronic kidney disease, low platelet count, and anemia, we relied on laboratory data.

#### Analysis or Efforts to Address Bias, Study Size, and Statistical Methods

Although we did not calculate an effect size a priori for this study, in our previous work, we have typically attempted to find a 5% or greater increase in anticoagulation initiation. A 5% increase would correspond with the prevention of 5 strokes over 1 year at our sites and 5000 strokes per year in the United States. We derive these figures from a large national registry reporting stroke rates in patients with AF as well as other epidemiological studies [22,23].

##### Message Opening, Website Review, and Survey-Based Outcomes

We calculated a chi-square-based *P* value comparing proportions of patients, or in the case of website site review, unique sessions, across messaging approaches or sites.

##### Anticoagulation Outcome

Among patients opening portal messages, we compared anticoagulation across the 2 messaging approaches. Specifically, we compared anticoagulation use 3 months after completion of messaging with both approaches for patients in group 1 and then separately for those in group 2. For this outcome, we excluded patients who did not have information to calculate baseline anticoagulation status (ie, visits within the past 12 months where anticoagulation status would have been updated) [12]. We did not impute missing anticoagulation status given the number of missing values and the unclear randomness of missingness as suggested in guidance from the literature [24]. To determine the significance of the difference in the percentage of anticoagulation use across message approaches, we calculated a chi-square-based *P* value, comparing proportions of anticoagulation separately for group 1 and then again for group 2.

To address potential bias from the confounder of the difference in populations at the 2 different sites, we computed the adjusted percentage of patients on anticoagulation between messaging approaches. More specifically, we constructed a generalized logistic mixed model with anticoagulation status (on or off) as the dependent variable and messaging approach (targeted vs blast) as the independent variable. We also included a random effect for provider to account for potential clustering and several covariates to adjust for potential confounders of anticoagulation. Covariates included variables making it more likely to be on anticoagulation (eg, higher stroke risk expressed through the CHA_2_DS_2_-VASc score) as well as factors making it less likely to be on anticoagulation (eg, anemia, chronic kidney disease, and high BMI). We did this separately for groups 1 and 2.

We performed all calculations in SAS (version 9.4; SAS Institute). In Multimedia Appendix 3, we include the code used to conduct the analysis.

### Ethical Considerations

At UMass, the institutional review board (IRB) approved this protocol with an implied consent process (ie, we argued consent would be implied by those choosing to review the website or answer our survey). We also provided patients with the opportunity to opt-out if they did not want us to use their information about message opening or anticoagulation use. At the time of analysis, all data were deidentified or anonymized by stripping real identifiers with a unique study identifier. All patients were informed before they provided implied informed consent. The UMass Chan IRB approved the waiver of documentation of written informed consent as the study was minimal-risk, appropriate confidentiality protections were to be exercised, and the waiver of consent would not adversely affect the rights and welfare of subjects. The authors designed the study and gathered and analyzed the data according to the Helsinki Declaration guidelines on human research. The research protocol used in this study was reviewed and approved by the UMass Chan IRB (H00021866). The authors did not use any form of AI in any portion of this study, including manuscript writing.

At UFL, the IRB exempted the study as quality improvement.

## Results

### Message Opening

We sent 1156 (UMass) and 668 (UFL) messages to group 1 patients, 438 and 632 messages to group 2 patients, and 92 and 150 messages to group 3 patients with the targeted and blast approaches, respectively. Cohort characteristics by group and messaging approach are described in Table 1.

Message opening was moderately high at both sites and across groups, with the highest opening rates in group 1 (723/1156, 62.5%) at UMass and group 3 (87/150, 57.3%) at UFL. Message opening in group 1 was significantly higher at UMass than at UFL (723/1156, 62.5% vs 382/668, 57.2%; *P*=.005). We did not find a statistically significant difference in message opening rates between the targeted (UMass) and blast (UFL) messaging approaches for group 2 (274/438, 62.6% vs 335/632, 53%; *P*=.09) and group 3 (52/92, 56.5% vs 86/150, 57.3%; *P*=.22).

**Table 1:** Key characteristics in patients receiving messages with targeted versus blast approach (percentages may not sum to 100 due to rounding).

Characteristics		Targeted messaging (University of Massachusetts)			Blast messaging (University of Florida)		
		Group 1 (high risk on anticoagulation; n=1156), n (%)	Group 2 (high risk off anticoagulation; n=438), n (%)	Group 3 (low risk; n=92), n (%)	Group 1 (high risk on anticoagulation; n=668), n (%)	Group 2 (high risk off anticoagulation; n=632), n (%)	Group 3 (low risk; n=150), n (%)
**Age**							
	<65	272 (23.5)	96 (21.9)	15 (16.3)	197 (29.5)	204 (32.3)	103 (68.7)
	65-74	374 (32.4)	140 (32)	44 (47.8)	256 (38.3)	192 (30.4)	40 (26.7)
	≥75	501 (43.3)	196 (44.8)	31 (33.7)	213 (31.9)	221 (35)	6 (4)
	Missing	9 (0.8)	6 (1.4)	2 (2.2)	2 (0.3)	15 (2.4)	1 (0.7)
**Sex**							
	Female	436 (37.7)	186 (42.5)	33 (35.9)	291 (43.6)	292 (46.2)	37 (24.7)
	Male	708 (61.2)	246 (56.2)	57 (62)	375 (56.1)	325 (51.4)	112 (74.7)
	Missing	12 (1)	6 (1.4)	2 (2.2)	2 (0.3)	15 (2.4)	1 (0.7)
**Race**							
	Black	18 (1.6)	4 (0.9)	0 (0)	181 (27.1)	121 (19.1)	225 (16.7)
	Other	49 (4.2)	19 (4.3)	2 (2.2)	37 (5.5)	29 (4.6)	5 (3.3)
	White	1080 (93.4)	414 (94.5)	90 (97.8)	447 (66.9)	463 (73.2)	119 (79.3)
	Decline to answer, missing, or unknown	9 (0.8)	1 (0.2)	0 (0)	3 (0.4)	19 (3)	1 (0.7)
**Ethnicity**							
	Hispanic or Latino	44 (3.8)	14 (3.2)	3 (3.3)	20 (3)	13 (2)	6 (4)
	Not Hispanic or Latino	1089 (94.2)	420 (95.9)	88 (95.6)	644 (96.4)	597 (94.5)	142 (94.7)
	Decline to Answer	22 (1.9)	4 (0.9)	1 (1.1)	1 (0.1)	2 (0.3)	0 (0)
	Unknown or missing	1 (0.1)	0 (0)	0 (0)	3 (0.4)	20 (3.2)	2 (1.3)
**Language preference**							
	English	1109 (95.9)	419 (95.7)	90 (97.8)	649 (97.2)	604 (95.6)	148 (98.7)
	Not English	47 (4.1)	19 (4.3)	2 (2.2)	16 (2.4)	11 (1.7)	2 (1.3)
	Unknown or missing	0 (0)	0 (0)	0 (0)	3 (0.4)	17 (2.7)	1 (0.7)

### Website Review

Using Google Analytics, we observed that few patients reviewed the Upbeat website—80 and 76 unique page views (*P=*.56) at UMass and UFL, respectively. For those that did review the website, the number that interacted with only viewed a single page of the website, that is, the “bounce rate” across both sites was between 54% and 57%. While a bounce rate of 40% or less is generally considered good [19], the referenced source did not provide a specific bounce rate for health education websites, which may differ from other types of websites. Bounce rates are best understood in the context of a website’s purpose and type. The average bounce rate for an informational website and landing pages tends to be higher than other website types [25], thus our findings indicate moderately high engagement with the Upbeat website.

The average session duration was shorter at UFL than at UMass (83 seconds vs 148 seconds). Although we can conduct a statistical test for the average session duration, Google Analytics did not provide the distribution of individual times for each unique page viewer (Table S1 in Multimedia Appendix 4 [19,26-29] for the remaining comparisons).

### Survey-Based Outcomes

From Group 1, 93 and 59 patients answered our survey using the targeted and blast messaging approaches, respectively. There was not a significant difference in patient reports of discussion with their provider about stroke risk, the duration of current anticoagulation use, or the frequency of missing doses of anticoagulation. Notably, forgetfulness and other reasons (apart from costs, side effects, or lack of benefit) comprise the majority of reasons for forgetting doses across messaging approaches. Most patients in both the targeted vs blast messaging groups strongly agreed or agreed that the materials from the HRS were easy-to-understand (68/82, 83% vs 13/15, 87%)*,* were useful (69/82, 84% vs 14/15, 93%), as well as something they would recommend (71/83, 85% vs 14/15, 93%) without any of the differences reaching statistical significance (Table S2 in Multimedia Appendix 4 [27] for details).

From Group 2, a total of 9/25 patients answered our survey using the targeted and blast messaging approaches, respectively. More patients in the UMass group had discussed their stroke risk with their physician at UMass (16/25, 64% vs 3/9, 33%; *P=*.04). Among the patients in the targeted approach, only 26% (6/25) reported concern about the risk of bleeding as a cause for stopping anticoagulation. Only 4 patients from the blast approach answered this item, limiting comparison. The majority of patients strongly agreed or agreed that the materials from the HRS were easy-to-understand and useful, as well as something they would recommend (also, comparisons were limited due to only 3 patients from the blast messaging group answering this item; Table S3 in Multimedia Appendix 4 [27]).

For group 3, we only had 2 responses from the targeted approach and 1 response from the blast approach and therefore did not conduct any further calculations or comparisons.

### Anticoagulation

For this outcome, we excluded patients for whom we did not have information to calculate baseline anticoagulation status (ie, visits within the past 12 months where anticoagulation status would have been updated) [12]. Among the included patients on anticoagulation (group 1) who opened messages, there were 636 and 285 from the targeted messaging and blast messaging approaches, respectively. Most patients reported race as White, with 91.8% (584/636) and 84.2% (240/285) under the targeted messaging and blast messaging approaches, respectively (Table 2).

The percentage of patients from group 1 on anticoagulation did not differ between targeted versus blast messaging approaches. By contrast, 11.9% (21/176) versus 3% (3/100; *P=*.01) of patients from group 2 in targeted versus blast messaging were on anticoagulation at the end of follow-up (Table 3). This difference persisted after adjustment with an anticoagulation percentage of 9.3% versus 2.1% (*P*<.001; Table 3).

**Table 2:** Key characteristics of patients opening messages with a targeted versus blast approach (percentages may not sum to 100 due to rounding).

Characteristics								Targeted messaging (University of Massachusetts; n=636), n (%)	Blast messaging (University of Florida; n=285), n (%)
**Age**									
	<65							60 (9.4)	9 (3.2)
	65-74							200 (31.4)	80 (28.1)
	≥75							376 (59.1)	196 (68.8)
**Sex**									
	Female							248 (39)	117 (41.1)
	Male							388 (61)	168 (58.9)
**Race^a^**									
	Black							9 (1.4)	28 (9.8)
	Hispanic							17 (2.7)	6 (2.1)
	Other							17 (2.7)	11 (3.8)
	White							584 (91.8)	240 (84.2)
	Missing							9 (1.4)	0 (0)
**Insurance**									
	Commercial							62 (9.7)	21 (7.4)
	Medicare							522 (82.1)	242 (84.9)
	Medicaid							20 (3.1)	0 (0)
	Other or state health insurance exchange							32 (5)	7 (2.4)
	Missing							0 (0)	15 (5.3)
**Anemia^b^**									
	Yes							359 (56.4)	146 (51.2)
	No							269 (42.3)	131 (46)
	Unknown							8 (1.3)	8 (2.8)
**Chronic kidney disease^c^**									
	Stage 1							162 (25.5)	63 (22.1)
	Stage 2							195 (30.7)	95 (33.3)
	Stage 3							219 (34.4)	101 (35.4)
	Stage 4 or 5							60 (9.4)	22 (7.7)
	Missing							0 (0)	4 (1.4)
**BMI Group**									
	Morbid obesity^d^							51 (8)	15 (5.3)
	Not morbidly obese							585 (92)	270 (94.7)
**Anticoagulant use at baseline**									
	Yes							459 (72.2)	186 (65.3)
	No							177 (27.8)	99 (34.7)
**Antiplatelet use**									
	Yes							353 (55.5)	237 (83.2)
	No							283 (44.5)	48 (16.8)

^a^Black includes Black of African American and multiracial, including Black or African American, Hispanic includes those individuals reporting Hispanic or Latino ethnicity, Asian includes White Hispanic. There were no individuals who reported Black race or Hispanic ethnicity. Other include Asian, Native American, Alaska Native, and others.

^b^Defined using established criteria, that is, hemoglobin <13 g/dL for male candidates and <12 g/dL for female candidates [30].

^c^Defined by established criteria [26] as a creatinine clearance calculated in mL/minute/1.73 m^2^ units for each stage: >90 (stage 1), 60-80 (stage 2), 30-59 (stage 3), 15-29 (stage 4), and <15 (stage 5).

^d^Obesity indicates a BMI ≥40.0 kg/m^2^, as defined by the World Health Organization [31].

**Table 3:** Unadjusted and adjusted percentages of anticoagulation for 2 messaging approaches or sites stratified by group.

	Unadjusted percentage on anticoagulation			Adjusted percentage on anticoagulation		
	Targeted messaging (UMass^a^), n (%)	Blast messaging (UFL^b^), n, (%)	*P* value^c^	Targeted messaging (UMass)^d^, %	Blast messaging (UFL)^d^, %	*P* value^e^
Group 1 (high risk, on anticoagulation at baseline)	438 (95.4)	179 (96.2)	.64	96.6	96.4	.52
Group 2 (high risk, off anticoagulation at baseline)	21 (11.9)	3 (3.0)	.01	9.3	2.1	<.001

^a^UMass: University of Massachusetts.

^b^UFL: University of Florida.

^c^Chi-square–based *P* value.

^d^Only percentages are shown.

^e^Value derived from generalized estimating equation adjusting for age, gender, BMI, patient race-ethnicity, insurance, CHA_2_DS_2_-VASc score, presence of anemia (ie, hemoglobin <13 g/dL for male candidates, <12 g/dL for female candidates, and level of chronic kidney disease).

## Discussion

### Principal Results

The message opening was significantly higher with the targeted approach for patients on anticoagulation. Subsequent website reviews were not different across approaches. Notably, 7.2% more patients off anticoagulation at baseline started anticoagulation with the targeted approach.

### Comparison With Previous Work

Several published studies have examined the impact of portal messaging. Toscos et al [32] and Toscos et al [33] found that a multicomponent intervention that included sending portal messages led to higher AF knowledge and adherence in AF patients randomized to the intervention compared to controls. The authors only focused on patients who had already been prescribed anticoagulation and found higher rates of patient portal use, similar to what we found in terms of message opening in this patient group. Szilagyi et al [34] demonstrated a small increase in influenza vaccination rates (on the order of 1%-3%) for patients receiving portal messages versus those not receiving one, but the authors did not study the delivery of the message in targeted versus blast approaches as we did. By contrast, Halket et al [35] studied the use of targeted electronic portal messaging for hepatitis C screening. More specifically, they studied the effect of sending a patient portal message for patients having an appointment in the upcoming 6 months compared with sending this message to those without an upcoming visit. Compared to controls, they found that 10% more patients (59/227, 26% vs 52/318, 16.4%; *P*<.01) underwent screening with the targeted approach. The authors do not further report the optimal timing within 6 months for sending a message. Presumably closer to the time of the visit would achieve the best results.

The main implication of this study is that targeted messaging was more effective than blast messaging in achieving message opening for those on anticoagulation. There was a trend toward increased message opening among patients off anticoagulation (274/438, 62.6% for the targeted approach versus 335/632, 53% for the blast approach). This increased message opening may have explained some anticoagulation starts, but replication at other sites would be helpful in drawing firm conclusions. Given the low rates of website reviews it is unlikely that it contributed to anticoagulation starts and would not be valuable to include in future programs, at least in the way we delivered it (as a simple website link). Education provided directly in the message or within the health portal is likely to be more effective than requesting patients to review external websites.

There are other implications for our findings. The best approach to messaging patients should also factor in local resources. Our approach to sending targeted messaging required the daily execution of a workbench report and subsequent filtering and transmission of portal messages. In the future, we anticipate that we could automate the manual steps and link the messaging with portal messages sent to patients related to preparation for ambulatory visits. Blast messaging may be successful in other contexts, such as for anticipated health programs such as yearly vaccination campaigns, as previously demonstrated. Although we sent blast messaging manually, automation could likely replace the manual process that we undertook and would likely require less support from IT professionals to code compared with targeted messaging. The clinical context, along with the cost and availability of IT support, should therefore dictate the approach that institutions and providers take when determining how to deliver messages to their patients.

### Limitations

We acknowledge several limitations of this proposed study. Most notably, we did not randomly allocate patients to messaging approaches. Each site pursued the approach of its preference. Thereby, baseline differences in populations and provider practice patterns may have explained some of our findings. This is especially true for the outcomes of message opening and website review, where we did not have patient-level variables. For the anticoagulation outcome, we adjusted for known confounders of the use of anticoagulation, including demographics, stroke risk score, and bleeding risk factors (ie, anemia and chronic kidney disease). Many other factors, including other indications for anticoagulation, type of anticoagulant, baseline health literacy, and computer literacy, may, however, have been different across our sites. In addition, because the site of care dictated the receipt of one versus the other messaging approach and we had limited information about the reason for receiving care at one versus the other site, we did not pursue propensity or other causal inference modeling approaches. Other institutional-based programs may have explained the increase with the targeted approach. At the same time, we were not aware of any systemwide programs at our sites during the time that we conducted this study. Additionally, we did not specifically test the messages with patients in a human-centered design approach. A human-centered design approach has successfully overcome limitations in other messaging programs cited in the literature [32,36]. Oake et al [36] observed that an automated voice messaging response system for communicating anticoagulation testing and dosage schedules to patients led to improved anticoagulation monitoring. Another limitation was that we were not able to ascertain if patients read our message, only that our portal message was opened. In many cases, a family member will be opening the message. Website review and survey responses may be limited in the same way. Although education by proxy through a family member may lead to decisions to take anticoagulation or stay on it, we were not able to distinguish the discrete effect of direct versus proxy communication in the current study. Our results may also not generalize to non-White populations, which is significant given the lower adherence of non-Whites [8]. Lastly, it is important to note the impact of COVID-19 and the timing of the UFL messages on the project. UFL messages were sent out in December, with a follow-up in January. In addition to patients receiving holiday-related emails, COVID-19 was surging as well. It is unclear how these two variables may have impacted message opening.

### Conclusion

In conclusion, message opening was significantly higher with the targeted approach for patients on anticoagulation. Subsequent website reviews were not different across approaches. More patients off anticoagulation at baseline started anticoagulation with the targeted approach. The best approach to messaging patients should also factor in local resources.

## Appendix

Multimedia Appendix 1 Patient portal messages that were sent to patients in Groups 1, 2, and 3.

Multimedia Appendix 2 Blank questionnaires that were completed by patients in Groups 1, 2, and 3.

Multimedia Appendix 3 SAS Code for Analysis of Anticoagulation Initiation Following Two Patient Portal Messaging Programs.

Multimedia Appendix 4 Supplemental Tables S1-S3.

## Abbreviations

AF: atrial fibrillation
CHA2DS2-VASc: congestive heart failure, hypertension, age ≥75 years, diabetes mellitus, stroke, vascular disease, age 65-74 years, sex scale
EHR: electronic health record
HRS: Heart Rhythm Society
IRB: institutional review board
UFL: University of Florida College of Medicine-Jacksonville
UMass: University of Massachusetts Chan Medical School

## Data Availability

The data sets generated during and/or analyzed during this study are available from the corresponding author on reasonable request.
