# Supplementary material for: Comparing the Efficacy of Targeted and Blast Portal Messaging in Message Opening Rate and Anticoagulation Initiation in Patients With Atrial Fibrillation in the Preventing Preventable Strokes Study II: Prospective Cohort Study
Source: JMIR Cardio. 2024 Jan 24;8:e49590. doi: 10.2196/49590 (PMC10851125; doi:10.2196/49590)
Supplement: Multimedia Appendix 1 [file cardio_v8i1e49590_app1.pdf]

Multimedia Appendix – MyChart Messages Sent to Patients

This is a Multimedia Appendix to a full manuscript authored by Kapoor et al published in the Journal of Medical Internet Research (JMIR) Cardio. For full copyright and citation information, please see http://dx.doi.org/10.2196/jmir.xxxx.

Patient Portal Message for Group 1 (at high risk, on AC)

You are receiving this communication as part of a research project UMass Medical School is conducting in partnership with the Heart Rhythm Society.

Your medical record indicates that you have been diagnosed with Atrial Fibrillation (AFib)/Atrial Flutter, types of irregular heartbeat (arrhythmia). Patients with AFib have an increased risk of stroke (5x greater than for those without the condition). That stroke risk is reduced by taking a blood thinner (also called an anticoagulant).

Your medical record shows that you currently have an active prescription for this type of medication.

Based on your recorded diagnosis of AFib and your risk factors for stroke, it is recommended that you continue this blood thinner unless instructed otherwise by your health care provider. We encourage you to discuss the benefits of taking a blood thinner at your upcoming appointment and tell your health care provider about any changes to your health.

We invite you to learn more about AFib, your risk factors for stroke, how you can decrease your risk of stroke, and the benefits of a blood thinner for patients with AFib by visiting the Heart Rhythm Society patient education website, UpBeat.org. Go to:

[LINK TO PROFESSIONAL SOCIETY MATERIALS]

We especially encourage you to select the “Learn More” links in the box on the right side of the screen.

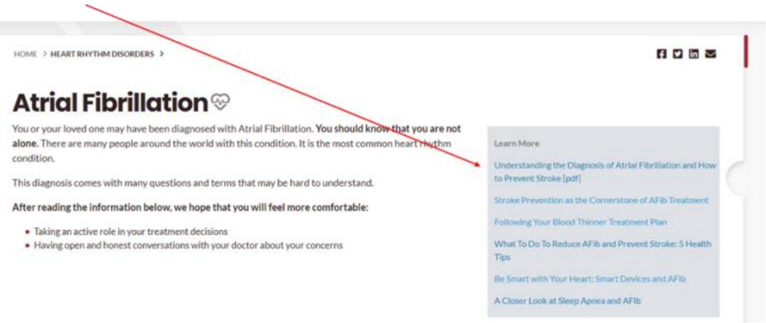

Your completion of the short survey at the following link would also be greatly appreciated:

[LINK TO REDCAP SURVEY]

Included in this message is a fact sheet explaining what it means to participate in the study, as well as a HIPAA form detailing what information we are requesting to conduct our study.

You do not have to do anything to sign up for the study. For more information on this research project, or if you would like to be excluded from the study, please contact our study team at [phone number].

Thank you from the Preventing Preventable Strokes Study Team!

Patient Portal Message for Group 2 (at risk, off AC)

You are receiving this communication as part of a research project UMass Medical School is conducting in partnership with the Heart Rhythm Society.

Your medical record indicates that you have been diagnosed with Atrial Fibrillation (AFib)/Atrial Flutter, types of irregular heartbeat (arrhythmia). Patients with AFib have an increased risk of stroke (5x greater than for those without the condition). That stroke risk is reduced by taking a blood thinner (also called an anticoagulant).

Your medical record shows that you do not have an active prescription for this type of medication.

Given that you have an upcoming appointment with a UMass Memorial Health cardiology or primary care provider, we wanted to help you prepare for this visit by reviewing the following information.

Based on your recorded diagnosis of AFib and your risk factors for stroke, you may want to discuss the benefits of taking a blood thinner at your upcoming appointment with your healthcare provider and decide if a blood thinner is right for you.

We invite you to learn more about AFib, your risk factors for stroke, how you can decrease your risk of stroke, and the benefits of a blood thinner for patients with AFib

by visiting the Heart Rhythm Society patient education website, UpBeat.org. Go to:

[LINK TO PROFESSIONAL SOCIETY MATERIALS]

We especially encourage you to select the “Learn More” links in the box on the right side of the screen.

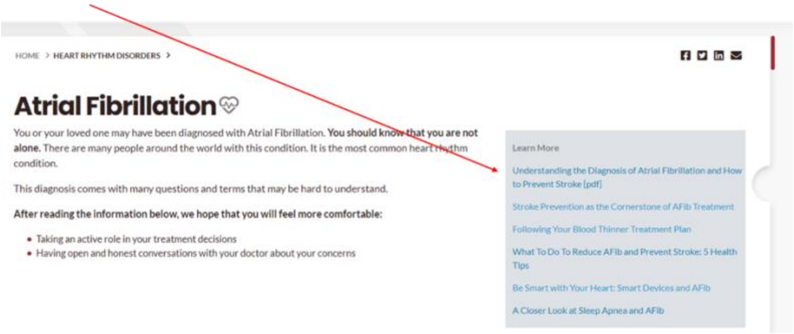

Your completion of the short survey at the following link would also be greatly appreciated:

[LINK TO REDCAP SURVEY]

Included in this message is a fact sheet explaining what it means to participate in the study, as well as a HIPAA form detailing what information we are requesting to conduct our study.

You do not have to do anything to sign up for the study. For more information on this research project, or if you would like to be excluded from the study, please contact our study team at [phone number].

Thank you from the Preventing Preventable Strokes Study Team!

Patient Portal Message for Group 3 (low risk, off AC)

You are receiving this communication as part of a research project UMass Medical School is conducting. We are partnering with the Heart Rhythm Society to increase patient awareness of the benefits of taking a blood thinner (also called an anticoagulant).. This medication reduces the risk of stroke in patients with Atrial Fibrillation (AFib)/Atrial Flutter which are types of irregular heart rhythm (arrhythmia).

However, currently you do NOT meet the criteria where this type of medication would be recommended.

The recommendation for a blood thinner is based on a patient’s age and risk of stroke. At your upcoming appointment, we encourage you to tell your health care provider about any changes to your health and discuss the benefits of a blood thinner for patients like you. At some point in the future, your health care provider may recommend your taking a blood thinner to help reduce stroke risk.

We invite you to learn more about AFib, your risk factors for stroke, how you can decrease your risk of stroke, and the benefits of taking a blood thinner for patients with AFib by visiting the Heart Rhythm Society patient education website, UpBeat.org. Go to:

[LINK TO PROFESSIONAL SOCIETY MATERIALS]

We especially encourage you to select the “Learn More” links in the box on the right side of the screen.

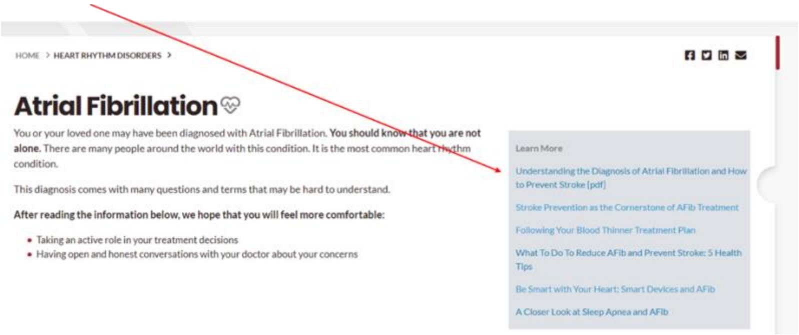

Your completion of the short survey at the following link would also be greatly appreciated:

[LINK TO REDCAP SURVEY]

Included in this message is a fact sheet explaining what it means to participate in the study, as well as a HIPAA form detailing what information we are requesting to conduct our study.

You do not have to do anything to sign up for the study. For more information on this research project, or if you would like to be excluded from the study, please contact our study team at [phone number].

Thank you from the Preventing Preventable Strokes Study Team!
