# Supplementary material for: Comparing the Efficacy of Targeted and Blast Portal Messaging in Message Opening Rate and Anticoagulation Initiation in Patients With Atrial Fibrillation in the Preventing Preventable Strokes Study II: Prospective Cohort Study
Source: JMIR Cardio. 2024 Jan 24;8:e49590. doi: 10.2196/49590 (PMC10851125; doi:10.2196/49590)
Supplement: Multimedia Appendix 2 [file cardio_v8i1e49590_app2.pdf]

Multimedia Appendix - Patient Surveys

This is a Multimedia Appendix to a full manuscript authored by Kapoor et al published in the Journal of Medical Internet Research (JMIR) Cardio. For full copyright and citation information, please see http://dx.doi.org/10.2196/jmir.xxxx.

PPS Questionnaire Group 1

Please complete the survey below.

Thank you!

Name ____________________

Have you ever discussed your risk of stroke with the health care provider you are scheduled to see next week?

- ◯ Yes
- ◯ No
- ◯ I am not seeing anyone next week
- ◯ Comment

Please specify "Comment" ____________________

How long have you been taking the blood thinner you are currently prescribed ?

Please note: blood thinner include warfarin (also known as coumadin), apixaban (also known as Eliquis), rivaroxaban (also known as Xarelto), enoxaparin (also known as Lovenox). Do not count aspirin or plavix.

- ◯ Less than 1 year
- ◯ 1-3 years
- ◯ More than 3 years
- ◯ I am not taking a blood thinner

How many days in the past week did you miss taking your blood thinner?

- ◯ 0 days
- ◯ 1 day
- ◯ 2 days
- ◯ 3 days
- ◯ 4 or more days
- ◯ I am not taking a blood thinner

When you do NOT take your blood thinner according to instructions, what are the reasons that you miss taking it?
Check as many as apply.

- ☐ Cost
- ☐ Side effects
- ☐ Forgetfulness
- ☐ Don't see benefit
- ☐ Other (please specify)
- ☐ I am not taking a blood thinner

Please specify "Other" ____________________

For the following statements, please rate your level of agreement.

	strongly disagree	disagree	neutral	agree	strongly agree
The Heart Rhythm Society educational materials were easy to understand	◯	◯	◯	◯	◯

The Heart Rhythm Society educational materials provide useful information	◯	◯	◯	◯	◯
I would recommend these materials to other patients interested in learning more about atrial fibrillation	◯	◯	◯	◯	◯

In the space below, kindly provide any additional information about what you liked or didn't like about the materials.

____

PPS Questionnaire Groups 2

Please complete the survey below.

Thank you!

Name ______________________________

Have you ever discussed your risk of stroke with the health care provider you are scheduled to see next week?
- ◯ Yes
- ◯ No
- ◯ Comment

Please specify "Comment" ______________________________

Has the health care provider you are scheduled to see next week ever recommended that you take a blood thinner?
- ◯ Yes, I took a blood thinner in the past
- ◯ Yes, but I did not take it
- ◯ No, we never discussed a blood thinner
- ◯ I don't remember

What would you say is the reason that most closely matches why you stopped taking your blood thinner?
- ☐ It was a temporary prescription
- ☐ My physician told me it was no longer necessary
- ☐ Concern about risk of bleeding
- ☐ Don't like taking medication
- ☐ Not concerned about risk of stroke
- ☐ No reason...I am still taking a blood thinner
- ☐ Other (please specify)

Please specify "Other" ______________________________

What has prevented you from taking your blood thinner? Check as many as apply.
- ☐ Cost
- ☐ Concern about risk of bleeding
- ☐ Don't like taking medication
- ☐ Not concerned about risk of stroke
- ☐ Other (please specify)

Please specify "Other" ______________________________

For the following statements, please rate your level of agreement.

	strongly disagree	disagree	neutral	agree	strongly agree
The Heart Rhythm Society educational materials were easy to understand	◯	◯	◯	◯	◯
The Heart Rhythm Society educational materials provide useful information	◯	◯	◯	◯	◯

I would recommend these materials to other patients interested in learning more about atrial fibrillation	○	○	○	○	○

In the space below, kindly provide any additional information about what you liked or didn't like about the materials.

____

PPS Questionnaire Group 3

Please complete the survey below.

Thank you!

Name ______________________________

How likely are you to spend time this year learning about your stroke risk

○ Not very likely ○ Not likely ○ Neutral ○ Somewhat likely ○ Very likely ○ Other (please specify)

Please specify "Other"

____

If your health care provider told you that you were now at an increased risk for stroke and could benefit from taking a blood thinner to decrease that risk, how likely would you be to take a blood thinner?

○ Not very likely ○ Not likely ○ Neutral ○ Somewhat likely ○ Very likely ○ Other (please specify)

Please specify "Other" ______________________________

What has prevented you from taking your blood thinner? Check as many as apply.

- ☐ Cost
- ☐ Concern about risk of bleeding
- ☐ Don't like taking medication
- ☐ Not concerned about risk of stroke
- ☐ Other (please specify)

Please specify "Other" ______________________________

For the following statements, please rate your level of agreement.

	strongly disagree	disagree	neutral	agree	strongly agree
The Heart Rhythm Society educational materials were easy to understand	○	○	○	○	○
The Heart Rhythm Society educational materials provide useful information	○	○	○	○	○
I would recommend these materials to other patients interested in learning more about atrial fibrillation	○	○	○	○	○

In the space below, kindly provide any additional information about what you liked or didn't like about the materials.

____
