# Supplementary material for: Comparing the Efficacy of Targeted and Blast Portal Messaging in Message Opening Rate and Anticoagulation Initiation in Patients With Atrial Fibrillation in the Preventing Preventable Strokes Study II: Prospective Cohort Study
Source: JMIR Cardio. 2024 Jan 24;8:e49590. doi: 10.2196/49590 (PMC10851125; doi:10.2196/49590)
Supplement: Multimedia Appendix 3 [file cardio_v8i1e49590_app3.pdf]

Multimedia Appendix

SAS Code for Analysis of Anticoagulation Initiation following Two Patient Portal Messaging Programs

This is a Multimedia Appendix to a full manuscript authored by Kapoor et al published in the Journal of Medical Internet Research (JMIR) Cardio. For full copyright and citation information, please see http://dx.doi.org/10.2196/jmir.xxxx.

```
OPTIONS MLOGIC MPRINT MERGENOBY=NOWARN /*USER='M:\Chen\PPS_Extension\Data'*/;

libname DS 'M:\Chen\PPS_Extension\SAS data';

libname UFLDS 'M:\Chen\PPS_Extension\UFL PPS\SAS data';


PROC FREQ DATA = DS.THIRD_FROMUSERID_NO_MISSING ;

        TABLE    ANTICOAGS_PRE patient_gender agegrp bmigrp antiplatelets raceeth  insurance_cat

                 cha2ds2vasc_cat anemia CKD_cons    / norow nocum;

        WHERE ANTICOAGS_PRE NE '' AND ANTICOAGS_POST NE '';

RUN;


PROC FREQ DATA = UFLDS.THIRD_FROMUSERID_NO_MISSING;

        TABLE    ANTICOAGS_PRE  patient_gender agegrp bmigrp antiplatelets raceeth  insurance_cat

                 cha2ds2vasc_cat anemia CKD_cons    / norow nocum;

        WHERE ANTICOAGS_PRE NE '' AND ANTICOAGS_POST NE '';

RUN;


PROC FREQ DATA = DS.THIRD_FROMUSERID_NO_MISSING;

        TABLE    (ANTICOAGS_PRE patient_gender agegrp bmigrp antiplatelets raceeth insurance_cat

                 cha2ds2vasc_cat anemia CKD_cons )*message_read / norow nopct;

        WHERE ANTICOAGS_PRE NE '' AND ANTICOAGS_POST NE '';

RUN;


PROC FREQ DATA = UFLDS.THIRD_FROMUSERID_NO_MISSING;

                 TABLE    (ANTICOAGS_PRE patient_gender agegrp bmigrp antiplatelets raceeth  insurance_cat2

                 cha2ds2vasc_cat anemia CKD_cons )*message_read / norow nopct;

WHERE ANTICOAGS_PRE NE '' AND ANTICOAGS_POST NE '';

RUN;
```

```
*Testing the unadjusted comparison of reading message effect on being on anticoagulation at the end of study ;

PROC FREQ DATA = DS.THIRD_FROMUSERID_NO_MISSING;

	TABLE	anticoags_pre*message_read*anticoags_post / nocol nopct chisq;

	WHERE ANTICOAGS_PRE NE '' AND ANTICOAGS_POST NE '';

RUN;

*testing found there was not significant effect although there was a trend for those not on AC at baseline to start AC but too small numbers;


*now look at the effect with adjustment first with interaction;

proc genmod data=DS.THIRD_FROMUSERID_NO_MISSING descending;

WHERE ANTICOAGS_PRE NE '' AND ANTICOAGS_POST NE '';

class ANTICOAGS_PRE message_read patient_gender agegrp bmigrp antiplatelets minority insurance_cat

			cha2ds2vasc_cat anemia CKD_cons assigned_PROVIDER;

Model ANTICOAGS_Post =  ANTICOAGS_PRE*message_read ANTICOAGS_PRE message_read

patient_gender agegrp bmigrp antiplatelets minority insurance_cat

			cha2ds2vasc_cat anemia CKD_cons / type3;

Repeated subject = assigned_PROVIDER / TYPE = IND;

LSMEANS ANTICOAGS_PRE*message_read / OM CL ILINK diff;

run;

*interaction is not positive;

*look at stratified effect of the intervention in those with _pre = yes vs no;

proc genmod data=DS.THIRD_FROMUSERID_NO_MISSING descending;

WHERE ANTICOAGS_PRE eq 'No' AND ANTICOAGS_POST NE '';

class ANTICOAGS_PRE message_read patient_gender agegrp bmigrp antiplatelets minority insurance_cat

			cha2ds2vasc_cat anemia CKD_cons assigned_PROVIDER;

Model ANTICOAGS_Post =  ANTICOAGS_PRE message_read

patient_gender agegrp bmigrp antiplatelets minority insurance_cat

			cha2ds2vasc_cat anemia CKD_cons;

Repeated subject = assigned_PROVIDER / TYPE = IND;

LSMEANS message_read / OM CL ILINK diff;

	ESTIMATE ' message read vs not read' message_read 1 -1 / EXP;
```

```
/*ESTIMATE 'GENDER Male vs Female' GENDER -1 1 / EXP;
ESTIMATE 'HL1_CONSOLIDATED Yes vs No' HL1_CONSOLIDATED -1 1 / EXP;
ESTIMATE 'HL2_CONSOLIDATED Yes vs No' HL2_CONSOLIDATED -1 1 / EXP;
ESTIMATE 'RANDOM1 Venous Indication vs Non-Venous Indication ' RANDOM1 -1 1 / EXP;
ESTIMATE 'RANDOM2 Yes vs No' RANDOM2 -1 1 / EXP;
ESTIMATE 'AC1 Yes vs No + all other categories' AC1 1 -1 / EXP;
ESTIMATE 'HS Yes vs No' HS -1 1 / EXP;
ESTIMATE 'MINORITY Yes vs No' MINORITY 1 -1 / EXP;
ESTIMATE 'AGE <65' AGE 1 0 0 -1 / EXP;
ESTIMATE 'AGE 65 - 74 vs < 75+' AGE 0 1 0 -1 / EXP;
ESTIMATE 'AGE 76+ vs < 50' AGE 0 0 1 -1 / EXP;
ESTIMATE 'INCOME 20-49,999 vs > 100,000' INCOME 1 0 0 0 -1 / EXP;
ESTIMATE 'INCOME 50-99,999 vs > 100,000' INCOME 0 1 0 0 -1 / EXP;
ESTIMATE 'INCOME < 20,000 vs > 100,000' INCOME 0 0 1 0 -1 / EXP;
ESTIMATE 'INCOME Prefer not to answer/Don’t know/Missing vs > 100,000' INCOME 0 0 0 1 -1 / EXP;
ESTIMATE 'AC_NAME2 Apixaban vs Not Apixaban' AC_NAME2 1 -1 / EXP;
ESTIMATE 'C_TOTAL_MEDICATIONS 11-15 vs 0-5' C_TOTAL_MEDICATIONS -1 1 0 0 0 / EXP;
ESTIMATE 'C_TOTAL_MEDICATIONS 16+ vs 0-5' C_TOTAL_MEDICATIONS -1 0 1 0 0 / EXP;
ESTIMATE 'C_TOTAL_MEDICATIONS 6-10 vs 0-5' C_TOTAL_MEDICATIONS -1 0 0 1 0 / EXP;
ESTIMATE 'C_TOTAL_MEDICATIONS Missing vs 0-5' C_TOTAL_MEDICATIONS -1 0 0 0 1 / EXP;
ESTIMATE 'CKD_CONS Missing vs Stage 1: Kidney damage with normal or increased GFR (>90 mL/min/1.73 m 2)' CKD_CONS 1 -1 0 0 0 / EXP;
ESTIMATE 'CKD_CONS Stage 2: Mild reduction in GFR (60-89 mL/min/1.73 m 2) vs Stage 1: Kidney damage with normal or increased GFR (>90 mL/min/1.73 m 2)' CKD_CONS 0 -1 1 0 0 / EXP;
ESTIMATE 'CKD_CONS Stage 3: Moderate reduction in GFR (30-59 mL/min/1.73 m 2) vs Stage 1: Kidney damage with normal or increased GFR (>90 mL/min/1.73 m 2)' CKD_CONS 0 -1 0 1 0 / EXP;
ESTIMATE 'CKD_CONS Stage 4/5: Severe reduction in GFR (15-29 mL/min/1.73 m 2)/Kidney failure (GFR < 15 mL/min/1.73 m 2 vs Stage 1: Kidney damage with normal or increased GFR (>90 mL/min/1.73 m 2)' CKD_CONS 0 -1 0 0 1 / EXP;
ESTIMATE 'ANEMIA Unknown vs No' ANEMIA -1 1 0/ EXP;
ESTIMATE 'ANEMIA Yes vs No' ANEMIA -1 0 1/ EXP; */
RUN;
proc genmod data=DS.THIRD_FROMUSERID_NO_MISSING descending;
WHERE ANTICOAGS_PRE eq 'Yes' AND ANTICOAGS_POST NE '';
```

```
class ANTICOAGS_PRE message_read patient_gender agegrp bmigrp antiplatelets minority insurance_cat
                    cha2ds2vasc_cat anemia CKD_cons assigned_PROVIDER;
Model ANTICOAGS_Post =  ANTICOAGS_PRE message_read
patient_gender agegrp bmigrp antiplatelets minority insurance_cat
                    cha2ds2vasc_cat anemia CKD_cons;
Repeated subject = assigned_PROVIDER / TYPE = IND;
LSMEANS message_read / OM CL ILINK diff;
          ESTIMATE ' message read vs not read' message_read 'yes' 'no' / EXP;
          /*ESTIMATE 'GENDER Male vs Female' GENDER -1 1 / EXP;
          ESTIMATE 'HL1_CONSOLIDATED Yes vs No' HL1_CONSOLIDATED -1 1 / EXP;
          ESTIMATE 'HL2_CONSOLIDATED Yes vs No' HL2_CONSOLIDATED -1 1 / EXP;
          ESTIMATE 'RANDOM1 Venous Indication vs Non-Venous Indication ' RANDOM1 -1 1 / EXP;
          ESTIMATE 'RANDOM2 Yes vs No' RANDOM2 -1 1 / EXP;
          ESTIMATE 'AC1 Yes vs No + all other categories' AC1 1 -1 / EXP;
          ESTIMATE 'HS Yes vs No' HS -1 1 / EXP;
          ESTIMATE 'MINORITY Yes vs No' MINORITY 1 -1 / EXP;
          ESTIMATE 'AGE <65' AGE 1 0 0 -1 / EXP;
          ESTIMATE 'AGE 65 - 74 vs < 75+' AGE 0 1 0 -1 / EXP;
          ESTIMATE 'AGE 76+ vs < 50' AGE 0 0 1 -1 / EXP;
          ESTIMATE 'INCOME 20-49,999 vs > 100,000' INCOME 1 0 0 0 -1 / EXP;
          ESTIMATE 'INCOME 50-99,999 vs > 100,000' INCOME 0 1 0 0 -1 / EXP;
          ESTIMATE 'INCOME < 20,000 vs > 100,000' INCOME 0 0 1 0 -1 / EXP;
          ESTIMATE 'INCOME Prefer not to answer/Don’t know/Missing vs > 100,000' INCOME 0 0 0 1 -1 / EXP;
          ESTIMATE 'AC_NAME2 Apixaban vs Not Apixaban' AC_NAME2 1 -1 / EXP;
          ESTIMATE 'C_TOTAL_MEDICATIONS 11-15 vs 0-5' C_TOTAL_MEDICATIONS -1 1 0 0 0 / EXP;
          ESTIMATE 'C_TOTAL_MEDICATIONS 16+ vs 0-5' C_TOTAL_MEDICATIONS -1 0 1 0 0 / EXP;
          ESTIMATE 'C_TOTAL_MEDICATIONS 6-10 vs 0-5' C_TOTAL_MEDICATIONS -1 0 0 1 0 / EXP;
          ESTIMATE 'C_TOTAL_MEDICATIONS Missing vs 0-5' C_TOTAL_MEDICATIONS -1 0 0 0 1 / EXP;
          ESTIMATE 'CKD_CONS Missing vs Stage 1: Kidney damage with normal or increased GFR (>90 mL/min/1.73 m 2)'
CKD_CONS 1 -1 0 0 0 / EXP;
          ESTIMATE 'CKD_CONS Stage 2: Mild reduction in GFR (60-89 mL/min/1.73 m 2) vs Stage 1: Kidney damage with
normal or increased GFR (>90 mL/min/1.73 m 2)' CKD_CONS 0 -1 1 0 0 / EXP;
```

```
        ESTIMATE 'CKD_CONS Stage 3: Moderate reduction in GFR (30-59 mL/min/1.73 m 2) vs Stage 1: Kidney damage
with normal or increased GFR (>90 mL/min/1.73 m 2)' CKD_CONS 0 -1 0 1 0 / EXP;

        ESTIMATE 'CKD_CONS Stage 4/5: Severe reduction in GFR (15-29 mL/min/1.73 m 2)/Kidney failure (GFR < 15
mL/min/1.73 m 2 vs Stage 1: Kidney damage with normal or increased GFR (>90 mL/min/1.73 m 2)' CKD_CONS 0 -1 0 0 1
/ EXP;

        ESTIMATE 'ANEMIA Unknown vs No' ANEMIA -1 1 0/ EXP;

        ESTIMATE 'ANEMIA Yes vs No' ANEMIA -1 0 1/ EXP; */

RUN;


*results from UMass above indicate a small trend towards message reading being associated with start of AC but no diff
with keeping patients on AC for those starting on AC at baseline;


*Now UfL data;

*Testing the unadjusted comparison of reading message effect on being on anticoagulation at the end of study ;

PROC FREQ DATA = UFLDS.THIRD_FROMUSERID_NO_MISSING;

        TABLE   anticoags_pre*message_read*anticoags_post / nocol nopct chisq;

        WHERE ANTICOAGS_PRE NE '' AND ANTICOAGS_POST NE '';

RUN;


PROC FREQ DATA = UFLDS.THIRD_FROMUSERID_NO_MISSING;

        TABLE   anticoags_pre*(message_read patient_gender agegrp bmigrp antiplatelets minority insurance_cat

                cha2ds2vasc_cat anemia CKD_cons) *anticoags_post / nocol nopct chisq;

        WHERE ANTICOAGS_PRE NE '' AND ANTICOAGS_POST NE '';

RUN;


*testing found there was not significant effect although there was a trend for those not on AC at baseline to start AC but
too small numbers;


*now look at the effect with adjustment first with interaction;

proc genmod data=UFLDS.THIRD_FROMUSERID_NO_MISSING descending;

WHERE ANTICOAGS_PRE NE '' AND ANTICOAGS_POST NE '';

class ANTICOAGS_PRE message_read patient_gender agegrp bmigrp antiplatelets minority insurance_cat

                cha2ds2vasc_cat anemia CKD_cons assigned_PROVIDER;
```

```
Model ANTICOAGS_Post =  ANTICOAGS_PRE*message_read ANTICOAGS_PRE message_read
patient_gender agegrp bmigrp antiplatelets minority insurance_cat
                        cha2ds2vasc_cat anemia CKD_cons / type3;
Repeated subject = assigned_PROVIDER / TYPE = IND;
LSMEANS ANTICOAGS_PRE*message_read / OM CL ILINK diff;
run;
*interaction is not positive;
*following regression does not converge - hessian;
proc genmod data=UFLDS.THIRD_FROMUSERID_NO_MISSING descending;
WHERE ANTICOAGS_PRE eq 'No' AND ANTICOAGS_POST NE '';
class ANTICOAGS_PRE message_read patient_gender agegrp bmigrp antiplatelets minority insurance_cat
                        cha2ds2vasc_cat anemia CKD_cons assigned_PROVIDER;
Model ANTICOAGS_Post =  ANTICOAGS_PRE message_read
patient_gender agegrp bmigrp antiplatelets minority insurance_cat
                        cha2ds2vasc_cat anemia CKD_cons;
Repeated subject = assigned_PROVIDER / TYPE = IND;
LSMEANS message_read / OM CL ILINK diff;
            ESTIMATE ' message read vs not read' message_read 1 -1 / EXP;
            /*ESTIMATE 'GENDER Male vs Female' GENDER -1 1 / EXP;
            ESTIMATE 'HL1_CONSOLIDATED Yes vs No' HL1_CONSOLIDATED -1 1 / EXP;
            ESTIMATE 'HL2_CONSOLIDATED Yes vs No' HL2_CONSOLIDATED -1 1 / EXP;
            ESTIMATE 'RANDOM1 Venous Indication vs Non-Venous Indication ' RANDOM1 -1 1 / EXP;
            ESTIMATE 'RANDOM2 Yes vs No' RANDOM2 -1 1 / EXP;
            ESTIMATE 'AC1 Yes vs No + all other categories' AC1 1 -1 / EXP;
            ESTIMATE 'HS Yes vs No' HS -1 1 / EXP;
            ESTIMATE 'MINORITY Yes vs No' MINORITY 1 -1 / EXP;
            ESTIMATE 'AGE <65' AGE 1 0 0 -1 / EXP;
            ESTIMATE 'AGE 65 - 74 vs < 75+' AGE 0 1 0 -1 / EXP;
            ESTIMATE 'AGE 76+ vs < 50' AGE 0 0 1 -1 / EXP;
            ESTIMATE 'INCOME 20-49,999 vs > 100,000' INCOME 1 0 0 0 -1 / EXP;
            ESTIMATE 'INCOME 50-99,999 vs > 100,000' INCOME 0 1 0 0 -1 / EXP;
            ESTIMATE 'INCOME < 20,000 vs > 100,000' INCOME 0 0 1 0 -1 / EXP;
```

```
	ESTIMATE 'INCOME Prefer not to answer/Don’t know/Missing vs > 100,000' INCOME 0 0 0 1 -1 / EXP;

	ESTIMATE 'AC_NAME2 Apixaban vs Not Apixaban' AC_NAME2 1 -1 / EXP;

	ESTIMATE 'C_TOTAL_MEDICATIONS 11-15 vs 0-5' C_TOTAL_MEDICATIONS -1 1 0 0 0 / EXP;

	ESTIMATE 'C_TOTAL_MEDICATIONS 16+ vs 0-5' C_TOTAL_MEDICATIONS -1 0 1 0 0 / EXP;

	ESTIMATE 'C_TOTAL_MEDICATIONS 6-10 vs 0-5' C_TOTAL_MEDICATIONS -1 0 0 1 0 / EXP;

	ESTIMATE 'C_TOTAL_MEDICATIONS Missing vs 0-5' C_TOTAL_MEDICATIONS -1 0 0 0 1 / EXP;

	ESTIMATE 'CKD_CONS Missing vs Stage 1: Kidney damage with normal or increased GFR (>90 mL/min/1.73 m 2)'
CKD_CONS 1 -1 0 0 0 / EXP;

	ESTIMATE 'CKD_CONS Stage 2: Mild reduction in GFR (60-89 mL/min/1.73 m 2) vs Stage 1: Kidney damage with
normal or increased GFR (>90 mL/min/1.73 m 2)' CKD_CONS 0 -1 1 0 0 / EXP;

	ESTIMATE 'CKD_CONS Stage 3: Moderate reduction in GFR (30-59 mL/min/1.73 m 2) vs Stage 1: Kidney damage
with normal or increased GFR (>90 mL/min/1.73 m 2)' CKD_CONS 0 -1 0 1 0 / EXP;

	ESTIMATE 'CKD_CONS Stage 4/5: Severe reduction in GFR (15-29 mL/min/1.73 m 2)/Kidney failure (GFR < 15
mL/min/1.73 m 2 vs Stage 1: Kidney damage with normal or increased GFR (>90 mL/min/1.73 m 2)' CKD_CONS 0 -1 0 0 1
/ EXP;

	ESTIMATE 'ANEMIA Unknown vs No' ANEMIA -1 1 0/ EXP;

	ESTIMATE 'ANEMIA Yes vs No' ANEMIA -1 0 1/ EXP; */

RUN;


proc genmod data=UFLDS.THIRD_FROMUSERID_NO_MISSING descending;

WHERE ANTICOAGS_PRE eq 'Yes' AND ANTICOAGS_POST NE '';

class ANTICOAGS_PRE message_read patient_gender agegrp bmigrp antiplatelets minority insurance_cat

		cha2ds2vasc_cat anemia CKD_cons assigned_PROVIDER;

Model ANTICOAGS_Post =  ANTICOAGS_PRE message_read

patient_gender agegrp bmigrp antiplatelets minority insurance_cat

		cha2ds2vasc_cat anemia CKD_cons;

Repeated subject = assigned_PROVIDER / TYPE = IND;

LSMEANS message_read / OM CL ILINK diff;

ESTIMATE ' message read vs not read' message_read 1 -1 / EXP;

	/*ESTIMATE 'GENDER Male vs Female' GENDER -1 1 / EXP;

	ESTIMATE 'HL1_CONSOLIDATED Yes vs No' HL1_CONSOLIDATED -1 1 / EXP;

	ESTIMATE 'HL2_CONSOLIDATED Yes vs No' HL2_CONSOLIDATED -1 1 / EXP;

	ESTIMATE 'RANDOM1 Venous Indication vs Non-Venous Indication ' RANDOM1 -1 1 / EXP;

	ESTIMATE 'RANDOM2 Yes vs No' RANDOM2 -1 1 / EXP;
```

```
	ESTIMATE 'AC1 Yes vs No + all other categories' AC1 1 -1 / EXP;
	ESTIMATE 'HS Yes vs No' HS -1 1 / EXP;
	ESTIMATE 'MINORITY Yes vs No' MINORITY 1 -1 / EXP;
	ESTIMATE 'AGE <65' AGE 1 0 0 -1 / EXP;
	ESTIMATE 'AGE 65 - 74 vs < 75+' AGE 0 1 0 -1 / EXP;
	ESTIMATE 'AGE 76+ vs < 50' AGE 0 0 1 -1 / EXP;
	ESTIMATE 'INCOME 20-49,999 vs > 100,000' INCOME 1 0 0 0 -1 / EXP;
	ESTIMATE 'INCOME 50-99,999 vs > 100,000' INCOME 0 1 0 0 -1 / EXP;
	ESTIMATE 'INCOME < 20,000 vs > 100,000' INCOME 0 0 1 0 -1 / EXP;
	ESTIMATE 'INCOME Prefer not to answer/Don’t know/Missing vs > 100,000' INCOME 0 0 0 1 -1 / EXP;
	ESTIMATE 'AC_NAME2 Apixaban vs Not Apixaban' AC_NAME2 1 -1 / EXP;
	ESTIMATE 'C_TOTAL_MEDICATIONS 11-15 vs 0-5' C_TOTAL_MEDICATIONS -1 1 0 0 0 / EXP;
	ESTIMATE 'C_TOTAL_MEDICATIONS 16+ vs 0-5' C_TOTAL_MEDICATIONS -1 0 1 0 0 / EXP;
	ESTIMATE 'C_TOTAL_MEDICATIONS 6-10 vs 0-5' C_TOTAL_MEDICATIONS -1 0 0 1 0 / EXP;
	ESTIMATE 'C_TOTAL_MEDICATIONS Missing vs 0-5' C_TOTAL_MEDICATIONS -1 0 0 0 1 / EXP;
	ESTIMATE 'CKD_CONS Missing vs Stage 1: Kidney damage with normal or increased GFR (>90 mL/min/1.73 m 2)' CKD_CONS 1 -1 0 0 0 / EXP;
	ESTIMATE 'CKD_CONS Stage 2: Mild reduction in GFR (60-89 mL/min/1.73 m 2) vs Stage 1: Kidney damage with normal or increased GFR (>90 mL/min/1.73 m 2)' CKD_CONS 0 -1 1 0 0 / EXP;
	ESTIMATE 'CKD_CONS Stage 3: Moderate reduction in GFR (30-59 mL/min/1.73 m 2) vs Stage 1: Kidney damage with normal or increased GFR (>90 mL/min/1.73 m 2)' CKD_CONS 0 -1 0 1 0 / EXP;
	ESTIMATE 'CKD_CONS Stage 4/5: Severe reduction in GFR (15-29 mL/min/1.73 m 2)/Kidney failure (GFR < 15 mL/min/1.73 m 2 vs Stage 1: Kidney damage with normal or increased GFR (>90 mL/min/1.73 m 2)' CKD_CONS 0 -1 0 0 1 / EXP;
	ESTIMATE 'ANEMIA Unknown vs No' ANEMIA -1 1 0/ EXP;
	ESTIMATE 'ANEMIA Yes vs No' ANEMIA -1 0 1/ EXP; */
RUN;

*results from UMass above indicate a small trend towards message reading being associated with start of AC but no diff with keeping patients on AC for those starting on AC at baseline;

PROC SQL;
	CREATE TABLE COMPARE_COL AS
```

```
	SELECT LIBNAME, NAME, TYPE, FORMAT
	FROM DICTIONARY.COLUMNS
	WHERE CATS(LIBNAME, ".", MEMNAME) IN ('DS.THIRD_FROMUSERID_NO_MISSING',
'UFLDS.THIRD_FROMUSERID_NO_MISSING') AND
			NAME IN ('ANTIPLATELETS', 'ANTICOAGS', 'EMOTIONAL_ABUSE', 'SEXUAL_ABUSE',
'PHYSICAL_ABUSE', 'PHYSICAL_ACTIVITY_DAYS_WEEK',
					'PHYSICAL_ACTIVITY_MIN_SESSION', 'PATIENT_HOMELESS',
'PATIENT_FAMILY_SIZE', 'PATIENT_FAMILY_INCOME')
	ORDER BY NAME, LIBNAME;
QUIT;


%MACRO REFORMAT;
	DATA _NULL_;
		SET COMPARE_COL (WHERE = (LIBNAME = 'UFLDS'));
		CALL SYMPUTX(CATS('V', _N_), NAME);
		CALL SYMPUTX(CATS('F', _N_), FORMAT);
	RUN;


	DATA REFORMAT_DATA (DROP = _C_:);
		FORMAT
		%DO I = 1 %TO 10;
			&&V&I &&F&I
		%END;
		;
		SET DS.THIRD_FROMUSERID_NO_MISSING (RENAME = (
			%DO I = 1 %TO 10;
				&&V&I = _C_&&V&I
			%END;
			));
		%DO I = 1 %TO 10;
			&&V&I = PUT(_C_&&V&I, BEST12.);
		%END;
	RUN;
```

```
%MEND REFORMAT;

%REFORMAT


DATA DS.COMBINED;

	LENGTH SITE $5. INSURANCE $46. LAST_CARD_VISIT_PROVIDER LAST_AMB_PROVIDER ASSIGNED_PROVIDER
$37. MYCHART_START_DATE $21. VETERAN_STATUS $19.;

	SET REFORMAT_DATA (IN = A) UFLDS.THIRD_FROMUSERID_NO_MISSING;

	IF A THEN SITE = 'Umass';

	ELSE SITE = 'Uflds';

	WHERE MESSAGE_READ = "Yes" AND ANTICOAGS_PRE NE '' AND ANTICOAGS_POST NE '';

RUN;

Data ds.combined;

set DS.COMBINED;

if insurance_cat='commercial' then insurance_cat2='commercial';

else if insurance_cat='medicare' then insurance_cat2='medicare';

else  insurance_cat2='other';

if ckd_cons='Missing' then CKD_cons2='Stage 1: Kidney damage with normal or increased GFR (>90 mL/min/1.73 m 2';

else ckd_cons2=ckd_cons;

if anemia='Unknown' then anemia2='No';

else anemia2=anemia;

run;


proc contents ;

run;

proc freq data=ds.combined;

WHERE ANTICOAGS_PRE eq 'No' ;

table (site patient_gender agegrp bmigrp /*antiplatelets*/ minority insurance_cat2

			cha2ds2vasc_cat anemia CKD_cons2)*anticoags_post;

run;

proc print;

*where ckd_cons='Missing';

var ckd_cons;
```

```
run;



*Comparing sites for AC starts and prevention of stops;
proc genmod data=ds.combined descending;
WHERE ANTICOAGS_PRE eq 'No' ;
class patient_gender agegrp bmigrp /*antiplatelets*/ minority insurance_cat2
			cha2ds2vasc_cat anemia2 CKD_cons2 assigned_PROVIDER SITE;
Model ANTICOAGS_Post =  SITE patient_gender agegrp bmigrp /*antiplatelets*/ minority insurance_cat2
			cha2ds2vasc_cat anemia2 CKD_cons2 ;
Repeated subject = assigned_PROVIDER / TYPE = IND;
LSMEANS site / OM CL ILINK diff;
/*ESTIMATE ' message read vs not read' message_read 1 -1 / EXP;
	ESTIMATE 'GENDER Male vs Female' GENDER -1 1 / EXP;
	ESTIMATE 'HL1_CONSOLIDATED Yes vs No' HL1_CONSOLIDATED -1 1 / EXP;
	ESTIMATE 'HL2_CONSOLIDATED Yes vs No' HL2_CONSOLIDATED -1 1 / EXP;
	ESTIMATE 'RANDOM1 Venous Indication vs Non-Venous Indication ' RANDOM1 -1 1 / EXP;
	ESTIMATE 'RANDOM2 Yes vs No' RANDOM2 -1 1 / EXP;
	ESTIMATE 'AC1 Yes vs No + all other categories' AC1 1 -1 / EXP;
	ESTIMATE 'HS Yes vs No' HS -1 1 / EXP;
	ESTIMATE 'MINORITY Yes vs No' MINORITY 1 -1 / EXP;
	ESTIMATE 'AGE <65' AGE 1 0 0 -1 / EXP;
	ESTIMATE 'AGE 65 - 74 vs < 75+' AGE 0 1 0 -1 / EXP;
	ESTIMATE 'AGE 76+ vs < 50' AGE 0 0 1 -1 / EXP;
	ESTIMATE 'INCOME 20-49,999 vs > 100,000' INCOME 1 0 0 0 -1 / EXP;
	ESTIMATE 'INCOME 50-99,999 vs > 100,000' INCOME 0 1 0 0 -1 / EXP;
	ESTIMATE 'INCOME < 20,000 vs > 100,000' INCOME 0 0 1 0 -1 / EXP;
	ESTIMATE 'INCOME Prefer not to answer/Don’t know/Missing vs > 100,000' INCOME 0 0 0 1 -1 / EXP;
	ESTIMATE 'AC_NAME2 Apixaban vs Not Apixaban' AC_NAME2 1 -1 / EXP;
	ESTIMATE 'C_TOTAL_MEDICATIONS 11-15 vs 0-5' C_TOTAL_MEDICATIONS -1 1 0 0 0 / EXP;
	ESTIMATE 'C_TOTAL_MEDICATIONS 16+ vs 0-5' C_TOTAL_MEDICATIONS -1 0 1 0 0 / EXP;
	ESTIMATE 'C_TOTAL_MEDICATIONS 6-10 vs 0-5' C_TOTAL_MEDICATIONS -1 0 0 1 0 / EXP;
```

```
ESTIMATE 'C_TOTAL_MEDICATIONS Missing vs 0-5' C_TOTAL_MEDICATIONS -1 0 0 0 1 / EXP;

ESTIMATE 'CKD_CONS Missing vs Stage 1: Kidney damage with normal or increased GFR (>90 mL/min/1.73 m 2)' CKD_CONS 1 -1 0 0 0 / EXP;

ESTIMATE 'CKD_CONS Stage 2: Mild reduction in GFR (60-89 mL/min/1.73 m 2) vs Stage 1: Kidney damage with normal or increased GFR (>90 mL/min/1.73 m 2)' CKD_CONS 0 -1 1 0 0 / EXP;

ESTIMATE 'CKD_CONS Stage 3: Moderate reduction in GFR (30-59 mL/min/1.73 m 2) vs Stage 1: Kidney damage with normal or increased GFR (>90 mL/min/1.73 m 2)' CKD_CONS 0 -1 0 1 0 / EXP;

ESTIMATE 'CKD_CONS Stage 4/5: Severe reduction in GFR (15-29 mL/min/1.73 m 2)/Kidney failure (GFR < 15 mL/min/1.73 m 2 vs Stage 1: Kidney damage with normal or increased GFR (>90 mL/min/1.73 m 2)' CKD_CONS 0 -1 0 0 1 / EXP;

ESTIMATE 'ANEMIA Unknown vs No' ANEMIA -1 1 0/ EXP;

ESTIMATE 'ANEMIA Yes vs No' ANEMIA -1 0 1/ EXP; */

RUN;



*Comparing sites for AC starts and prevention of stops;

proc genmod data=ds.combined descending;

WHERE ANTICOAGS_PRE eq 'Yes' ;

class patient_gender agegrp bmigrp /*antiplatelets*/ minority insurance_cat2

        cha2ds2vasc_cat anemia2 CKD_cons2 assigned_PROVIDER SITE;

Model ANTICOAGS_Post =  SITE patient_gender agegrp bmigrp /*antiplatelets*/ minority insurance_cat2

        cha2ds2vasc_cat anemia2 CKD_cons2 ;

Repeated subject = assigned_PROVIDER / TYPE = IND;

LSMEANS site / OM CL ILINK diff;

/*ESTIMATE ' message read vs not read' message_read 1 -1 / EXP;

    ESTIMATE 'GENDER Male vs Female' GENDER -1 1 / EXP;

    ESTIMATE 'HL1_CONSOLIDATED Yes vs No' HL1_CONSOLIDATED -1 1 / EXP;

    ESTIMATE 'HL2_CONSOLIDATED Yes vs No' HL2_CONSOLIDATED -1 1 / EXP;

    ESTIMATE 'RANDOM1 Venous Indication vs Non-Venous Indication ' RANDOM1 -1 1 / EXP;

    ESTIMATE 'RANDOM2 Yes vs No' RANDOM2 -1 1 / EXP;

    ESTIMATE 'AC1 Yes vs No + all other categories' AC1 1 -1 / EXP;

    ESTIMATE 'HS Yes vs No' HS -1 1 / EXP;

    ESTIMATE 'MINORITY Yes vs No' MINORITY 1 -1 / EXP;

    ESTIMATE 'AGE <65' AGE 1 0 0 -1 / EXP;
```

```
ESTIMATE 'AGE 65 - 74 vs < 75+' AGE 0 1 0 -1 / EXP;

ESTIMATE 'AGE 76+ vs < 50' AGE 0 0 1 -1 / EXP;

ESTIMATE 'INCOME 20-49,999 vs > 100,000' INCOME 1 0 0 0 -1 / EXP;

ESTIMATE 'INCOME 50-99,999 vs > 100,000' INCOME 0 1 0 0 -1 / EXP;

ESTIMATE 'INCOME < 20,000 vs > 100,000' INCOME 0 0 1 0 -1 / EXP;

ESTIMATE 'INCOME Prefer not to answer/Don't know/Missing vs > 100,000' INCOME 0 0 0 1 -1 / EXP;

ESTIMATE 'AC_NAME2 Apixaban vs Not Apixaban' AC_NAME2 1 -1 / EXP;

ESTIMATE 'C_TOTAL_MEDICATIONS 11-15 vs 0-5' C_TOTAL_MEDICATIONS -1 1 0 0 0 / EXP;

ESTIMATE 'C_TOTAL_MEDICATIONS 16+ vs 0-5' C_TOTAL_MEDICATIONS -1 0 1 0 0 / EXP;

ESTIMATE 'C_TOTAL_MEDICATIONS 6-10 vs 0-5' C_TOTAL_MEDICATIONS -1 0 0 1 0 / EXP;

ESTIMATE 'C_TOTAL_MEDICATIONS Missing vs 0-5' C_TOTAL_MEDICATIONS -1 0 0 0 1 / EXP;

ESTIMATE 'CKD_CONS Missing vs Stage 1: Kidney damage with normal or increased GFR (>90 mL/min/1.73 m 2)' CKD_CONS 1 -1 0 0 0 / EXP;

ESTIMATE 'CKD_CONS Stage 2: Mild reduction in GFR (60-89 mL/min/1.73 m 2) vs Stage 1: Kidney damage with normal or increased GFR (>90 mL/min/1.73 m 2)' CKD_CONS 0 -1 1 0 0 / EXP;

ESTIMATE 'CKD_CONS Stage 3: Moderate reduction in GFR (30-59 mL/min/1.73 m 2) vs Stage 1: Kidney damage with normal or increased GFR (>90 mL/min/1.73 m 2)' CKD_CONS 0 -1 0 1 0 / EXP;

ESTIMATE 'CKD_CONS Stage 4/5: Severe reduction in GFR (15-29 mL/min/1.73 m 2)/Kidney failure (GFR < 15 mL/min/1.73 m 2 vs Stage 1: Kidney damage with normal or increased GFR (>90 mL/min/1.73 m 2)' CKD_CONS 0 -1 0 0 1 / EXP;

ESTIMATE 'ANEMIA Unknown vs No' ANEMIA -1 1 0/ EXP;

ESTIMATE 'ANEMIA Yes vs No' ANEMIA -1 0 1/ EXP; */

RUN;


proc freq data=ds.combined;

WHERE ANTICOAGS_PRE eq 'Yes' ;

table site*ANTICOAGS_Post / chisq;

run;

proc freq data=ds.combined;

WHERE ANTICOAGS_PRE eq 'No' ;

table site*ANTICOAGS_Post / chisq;

run;
```
