# Supplementary material for: Comparing the Efficacy of Targeted and Blast Portal Messaging in Message Opening Rate and Anticoagulation Initiation in Patients With Atrial Fibrillation in the Preventing Preventable Strokes Study II: Prospective Cohort Study
Source: JMIR Cardio. 2024 Jan 24;8:e49590. doi: 10.2196/49590 (PMC10851125; doi:10.2196/49590)
Supplement: Multimedia Appendix 4 [file cardio_v8i1e49590_app4.pdf]

Multimedia Appendix – Supplemental Tables

This is a Multimedia Appendix to a full manuscript authored by Kapoor et al published in the Journal of Medical Internet Research (JMIR) Cardio. For full copyright and citation information, please see http://dx.doi.org/10.2196/jmir.xxxx.

Table S1. Comparison of Engagement Metrics across Two Messaging Approaches/Sites

Engagement Metric	Targeted Messaging (University of Massachusetts)	Blast Messaging (University of Florida)	*P*-value[a]
**Unique pageviews[b]**	4.8% (80 of 1672)	5.2% (76 of 1450)	.56
**Average Session Duration (in seconds)**	148	83	
	Frequency (% of 80)	Frequency (% of 76)	
**Bounce Rate[c]**	43 (54)	43 (57)	.72
**Interactions reported as frequency (% out of a number of unique page views)**			
YouTube video with cartoon informational video[27]	10 (13)	16 (21)	.21
Atrial fibrillation information sheets[28]	11 (14)	5 (7)	.04
All others combined (taxonomy cards[d], etc.)	3 (4)	6 (8)	.36

[a]Chi square-based *P*-value reported for metrics for which we could compare proportions. We did not have individual-level data available on session duration to compare means.

[b]The unique pageviews value represents the total number of unique sessions; we presumed that each session corresponded with one message being sent.[25]

[c]Bounce rate represents the percentage of all sessions on a site in which users only viewed a single page; an excellent bounce rate is 40% or less,[18] The referenced source did not provide a specific bounce rate for health education websites which may differ from other types of websites.

[d]Taxonomy cards represent information grouped by topic (treatments, heart rhythm disorders, early warning signs, etc.).[26]

Table S2: Survey Responses for Patients on AC (Group 1) across Two Messaging Approaches

Metric	Targeted Messaging (University of Massachusetts)	Blast Messaging (University of Florida)	*P*-value[a]
**UMass: Have you discussed stroke risk with the provider you are scheduled to see next week? UFL: Have you discussed your risk of stroke with your physician?**	Frequency (% of 93)	Frequency (% of 58)	
Yes	58 (62)	47 (81)	
No	22 (24)	10 (17)	.17[b]
Other (not having appointment/other comment)	13 (14)	1 (2)	N/A
**Duration of current anticoagulant use**	Frequency (% of 94)	Frequency (% of 59)	
Less than 1 year	18 (19)	4 (7)	N/A
1–3 years	37 (39)	22 (37)	
More than 3 years	39 (42)	29 (49)	.54[c]
Other (not taking AC)	0	4 (7)	N/A
**How many days in the past week did you miss taking your blood thinner?**	Frequency (% of 94)	Frequency (% of 55)	
0 days	83 (88)	50 (91)	.62
1 day	4 (4)	4 (7)	
2 or more days	7 (7)	1 (2)	N/A
**Reasons for missing blood thinner**	Frequency (% of 58)	Frequency (% of 44)	N/A[d]
Cost	4 (7)	0	
Side effects	0	0	
Forgetfulness	31 (53)	15 (34)	
Don't see benefit	0	0	
I am not taking a blood thinner	0	0	
Other (other comment)	26 (45)	31 (71)	
**Agreement[e] with statements about Heart Rhythm Society materials[f]**	Frequency (%)	Frequency (%)	
Materials were easy to understand	68 of 82 (83)	13 of 15 (87)	.72
Materials were useful	69 of 82 (84)	14 of 15 (93)	.35
I would recommend HRS materials to other patients	71 of 83 (85)	14 of 15 (93)	.41
**Did you visit UpBeat.org to learn more about the risk of stroke for people with AFib? (UFL only)**		Frequency (% of 58)	
Yes		16 (28)	
No, but I do intend to visit the site later		35 (60)	
No, and I do not intend to visit the site		5 (9)	N/A

Abbreviations: N/A = not applicable; used to indicate that a *P*-value was not obtained for categories with counts less than 5.

[a]All *P*-values were calculated by Chi-squared test.

[b]The Chi-squared test *P*-value calculated using only the "Yes" and "No" categories (rows).

[c]The Chi-squared test *P*-value calculated using only the "1 to 3 years" and "More than 3 years" categories.

[d]Given that patients could select more than one option for this item, we did not conduct Chi-squared testing.

[e]Included individuals who agreed or strongly agreed with the statement on a 5-point, Likert scale.

[f]Materials housed on Heart Rhythm Society's UpBeat.org website for atrial fibrillation[26] and included information that patients could review, download, and in some cases, watch.

Table S3. Survey Responses for Patients Not on AC (Group 2) across Two Messaging Approaches

Metric	Targeted Messaging (UMass)	Blast Messaging (UFL)	*P*-value[a]
**UMass: Have you discussed stroke risk with the provider scheduled to see next week? UFL: Have you discussed your risk of stroke with your physician?**	Frequency (% of 25)	Frequency (% of 9)	
Yes	16 (64)	3 (33)	N/A
No	7 (28)	6 (67)	.04
Other (not having appointment/other comment)	2 (8)	0	N/A
**UMass: Has the healthcare provider you are scheduled to see next week ever recommended that you take a blood thinner? UFL: Has your physician ever recommended that you take a blood thinner?**	Frequency (% of 22)	Frequency (% of 9)	
Yes, I took a blood thinner in the past	7 (32)	4 (44)	
Yes, but I did not take it	4 (18)	1 (11)	
No	10 (46)	2 (22)	N/A
Other (I don't remember)	1 (5)	2 (22)	
**What would you say is the reason that most closely matches why you stopped taking your blood thinner?**	Frequency (% of 23)	Frequency (% of 4)	
It was a temporary prescription	2 (9)	0	
My physician told me it was no longer necessary	5 (22)	0	
Concern about risk of bleeding	6 (26)	0	
Don't like taking medication	2 (9)	0	
Not concerned about risk of stroke	1 (4)	0	
No reason...I am still taking a blood thinner	3 (13)	1 (25)	
Other (other comment)	7 (30)	3 (75)	N/A[b]
**What has prevented you from taking your blood thinner?**	Frequency (% of 24)	Frequency (% of 1)	
Cost	1 (4)	0	
Side effects	7 (29)	0	
Don't like taking medication	2 (8)	0	N/A[b]
Not concerned about risk of stroke	5 (21)	0	
Other	13 (54)	1 (100)	
**Agreement[c] with statements about Heart Rhythm Society materials[d]**	Frequency (% of 21)	Frequency (% of 3)	N/A
Materials were easy to understand	14 (67)	3 (100)	
Materials were useful	15 (71)	3 (100)	
I would recommend HRS materials to other patients	15 (71)	3 (100)	
**After reviewing this material, do you plan to follow-up with your provider? (UFL only)**		Frequency (% of 9)	
Definitely		9 (100)	
Other (Maybe, No, comment)		0	N/A

**Did you visit UpBeat.org to learn more about the risk of stroke for people with AFib? (UFL only)**		Frequency (% of 9)	
Yes		3 (33)	
No, but I do intend to visit the site later		4 (44)	
No, and I do not intend to visit the site		2 (22)	N/A

Abbreviations: UMass = University of Massachusetts Chan School of Medicine; UFL = University of Florida College of Medicine – Jacksonville; N/A = not applicable, used to indicate that a *P*-value was not obtained for categories with counts less than 5.

[a]All *P*-values were calculated by Chi-squared test.

[b]Given that patients could select more than one option for this item, we did not conduct Chi-squared testing.

[c]Included individuals who agreed or strongly agreed with the statement on a 5-point, Likert scale.

[d]Materials housed on Heart Rhythm Society's UpBeat.org website for atrial fibrillation[26] and included information that patients could review, download, and in some cases, watch.
